# Redefining telemedicine in obstructive sleep apnea management through artificial intelligence

**DOI:** 10.3389/frsle.2025.1678077

**Published:** 2025-11-19

**Authors:** Ding Zou, Daniil Lisik, Sébastien Bailly, Johan Verbraecken

**Affiliations:** 1 Center for Sleep and Vigilance Disorders, Sahlgrenska Academy, University of Gothenburg, Gothenburg, Sweden; 2 The OLIN Unit, Section of Sustainable Health, Department of Public Health and Clinical Medicine, Umeå University, Umeå, Sweden; 3 Krefting Research Centre, Institute of Medicine, Sahlgrenska Academy, University of Gothenburg, Gothenburg, Sweden; 4 Grenoble Alps University, HP2 Laboratory, Inserm U1300, Grenoble Alps Hospital, Grenoble, France; 5 Antwerp University Hospital and University of Antwerp, Pulmonary Medicine and Multidisciplinary Sleep Disorders Centre, Antwerp, Belgium

**Keywords:** adherence, digital pathway, home sleep apnea test, patient centered care, positive airway pressure, precision medicine, treatable trait, wearable

## Abstract

Obstructive sleep apnea (OSA) represents a significant and increasingly prevalent health burden, impacting individual patients through diminished quality of life, increased morbidity and mortality, as well as society at large, via reduced productivity and escalating healthcare and welfare expenditures. As a multifactorial and heterogeneous disorder, OSA encompasses diverse endotypes and phenotypes, necessitating personalized approaches to diagnosis and management in order to achieve optimal clinical outcomes. Modern telemedicine encompasses a broad spectrum of digital tools designed to enhance the efficiency and precision of care delivery for complex conditions. Recent years have witnessed the rapid integration of advanced telehealth technologies, including consumer-grade devices, into clinical practice. Simultaneously, artificial intelligence (AI) has emerged as a transformative force in healthcare, enabling the automation of routine tasks, advanced data analytics, and the generation of novel clinical hypotheses. Within this domain, large language models, a subclass of AI specializing in natural language processing, offer new opportunities for augmenting patient-provider interactions, including streamlining communication and triaging patient-reported data. Despite these technological advancements, the full potential of telemedicine in the management of OSA remains underexplored. However, its implementation is expanding, particularly in longitudinal care models involving large patient cohorts. This Perspective aims to synthesize current state-of-the-art developments and proposes a comprehensive, integrated framework that leverages telemedicine, AI, and a multidimensional understanding of comorbidities and treatable traits throughout the continuum of OSA care, from screening and diagnosis to adherence monitoring and treatment optimization.

## Introduction

Obstructive sleep apnea (OSA) is a highly prevalent chronic condition. The global prevalence of OSA (by the definition of apnea-hypopnea index [AHI] ≥5 events per hour) in adults aged 30–69 years has been estimated at around 1 billion, with almost half of these having moderate/severe disease (AHI ≥15 events per hour). In Europe, such as France and Germany, the proportion of adults with OSA exceeds 50% (Benjafield et al., 2019). Recent decades have seen a sharp increase in diagnosed cases. For example, from 1996 to 2018, the annual number of treated sleep apnea patients in Finland has increased six-fold (Mattila et al., 2022). There are also indications that a further increase of OSA will occur (Boers et al., 2025), particularly in developing regions, where underdiagnosis is most pronounced (Iannella et al., 2025). Beyond being common, OSA inflicts substantial hardship and socioeconomic burden, through morbidity, productivity loss, impaired quality of life, and higher risk of mortality (Iannella et al., 2025; Wang et al., 2025). As with prevalence, the societal burden of OSA will likely also increase, partly due to the ongoing obesity epidemic (Finkelstein et al., 2012) (although leveling off has been reported in developed countries) (Koliaki et al., 2023) and an aging population, both common risk factors for OSA (de Araujo Dantas et al., 2023), but also due to external factors, such as global warming, through negative impact on sleep and OSA severity (Lechat et al., 2025).

**Figure 1:**
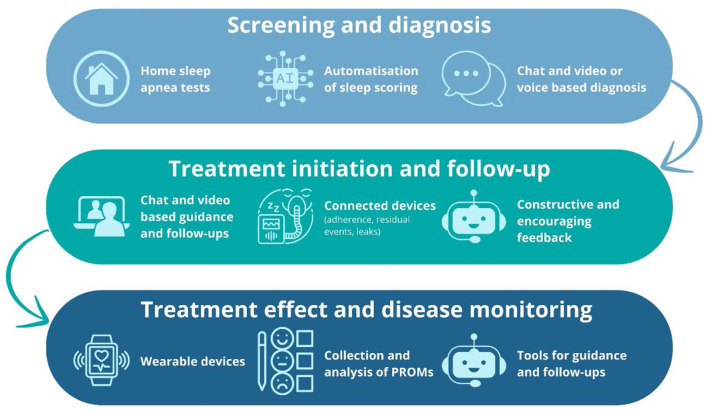
Digital pathway of telemedicine-based obstructive sleep apnea management, illustrating the integration of screening and diagnosis, treatment initiation and follow-up, and ongoing treatment effect and disease monitoring through home-based testing, AI-driven tools, telehealth, connected devices, and wearable technologies. PROM, patient-reported outcome.

The strain on the healthcare system has partly been met with restructuring treatment protocols. An indication of this can be seen in Finland, with a marked increase in 2005–2019 in remote care and nurse visits related to OSA but not outpatient physician visits (Peuranheimo et al., 2025). Telemedicine (TM; remote delivery of care through the use of technology) (Reid, 1996) is a common denominator in these efforts. TM has been around, in its most rudimentary forms, since the mid-1990s (Shiomi, 2000), but implementation within OSA has only really taken off in the past decade or so (Fietze et al., 2022b). A report from the European Sleep Apnea Database (ESADA), shows that in 2020, TM was used in an estimated 8% of diagnosis, 50% of treatment, and 73% of follow-up of OSA patients in (participating) European sleep centers (Fietze et al., 2022b). Despite a rapid increase in implementation, there appears to be potential to further increase these numbers and most importantly optimize the extant applications. With recent advent in sophisticated artificial intelligence (AI), including large language models (LLMs), the horizon of possibilities is extended even more (Oks et al., 2025). In this perspective, we provide an overview of the current state of art of TM in OSA management, from diagnosis to subtyping to treatment follow-up and risk management (Figure 1). Several reviews have been previously published on this topic (Verbraecken, 2021; Bailly et al., 2024; Pépin et al., 2025), but this work provides a more directed focus on incorporating AI and multidimensional treatable traits (TTs)—based management, extending beyond the often-seen “one-size-fits-all” positive airway pressure (PAP)-centric treatment strategy (McDonald and Holland, 2024).

## Primer on telemedicine (TM) and artificial intelligence (AI)

TM is defined as the provision of healthcare services and medical information using innovative technologies in situations where the health professional and patient (or two health professionals) are not in the same location (Lupiáñez-Villanueva et al., 2021). Despite its “tele-“ prefix, suggesting care delivered through telephone-based technologies, TM is currently constituted of a wide range of other devices, including wearables (e.g., smartwatches), nearables (e.g., mattress sensors), and various other internet of things (IoT) devices (Yoon and Choi, 2023). These devices can transmit virtually any type of information, both “objectively” collected parameters, such as blood oxygenation, as well as subjectively reported information, such as patient feedback. In terms of methods of relaying information, TM has classically been divided into *synchronous* (real-time transmission and assessment of data and communication) and *asynchronous* (transmission and storage of data for later assessment) (Kuziemsky et al., 2019).

A relatively novel component in TM is AI. The rationale of incorporating AI is largely to automate or streamline time-consuming/repetitive processes. AI, particularly the subset of AI called machine learning (ML; i.e., algorithms/models that learn patterns from data without explicit programming/instructions) (Sarker, 2021), is suitable for such applications (Kuziemsky et al., 2019). Furthermore, AI can be used to explore complex data, including multimodal and/or longitudinal dimensions of data, and generate hypotheses, for example regarding novel disease subtypes (Zinchuk et al., 2017).

Although ML and AI are rapidly expanding in medicine, their application to telemonitoring remains limited. A first example of AI use in PAP therapy concerns the prediction of adherence, which is a major worldwide issue given the persistently low adherence rates and high rates of PAP discontinuation over time (Pepin et al., 2021a). In this context, AI approaches such as random forest models can be applied at treatment initiation to identify the most appropriate device settings based on baseline features, including heart rate variability and oximetry-derived parameters (Kuo et al., 2025). Several ML approaches have been compared for predicting early PAP adherence (Rafael-Palou et al., 2018; May and Dalton, 2024; Scioscia et al., 2022), showing that algorithms perform reasonably well in predicting patients at the extremes (i.e., highly adherent vs. poorly adherent) and in exploring factors associated with poor adherence (Eguchi et al., 2022). However, most of these approaches rely on static or limited longitudinal datasets rather than fully dynamic data streams. Incorporating continuously collected telemonitoring data (e.g., nightly usage patterns, mask leak trajectories, residual respiratory events) into predictive models would allow the detection of early warning signals, such as a sudden decline in adherence after a few nights or a progressive deterioration in mask fit. Such dynamic approaches could trigger timely, targeted interventions before treatment failure becomes established. Beyond adherence prediction, AI tools embedded in connected devices can also enhance remote PAP management by integrating, for example, patient-reported outcomes (PROMs) collected by short questionnaires or environmental data collected by sensors, ultimately transforming telemonitoring from a passive reporting tool into an active, adaptive system of care.

## Screening and diagnosis

Diagnosis of OSA traditionally relies on monitored in-laboratory over-night polysomnography (PSG; level I test) (Ferber et al., 1994). This type of examination is costly and burdensome, requiring sleeping in an unfamiliar environment. Home sleep apnea testing (HSAT) has been proposed as a viable alternative in the diagnostic toolbox. HSAT options range from ambulatory PSG (level II test) (Zou et al., 2006), self-applied PSG (Ferretti et al., 2025), and level III cardiorespiratory polygraphy (Xu et al., 2017), to limited-channel studies (level IV test) (Bailer et al., 2024). With the advent of novel AI technologies, some level III devices are now capable of assessing total sleep time and arousals via a respiratory inductance plethysmography signal (Leger and Elbaz, 2024; Finnsson et al., 2025), allowing for more accurate AHI calculation (level III plus device). Additionally, some screening devices (level III equivalent device) that utilize advanced sensors and ML technology have demonstrated good validity of respiratory event index when compared to standard PSG derived AHI (sensitivity 0.92, specificity 0.84 for AHI≥15 event/h) (Pepin et al., 2020). Some screening devices also support multi-night testing, which can enhance the accuracy of OSA diagnosis (Zou et al., 2023).

Diagnosing OSA is a multi-step process, and TM may streamline most of the steps. First, AI (not least capable LLMs), may help in history-taking (Kuziemsky et al., 2019), which could shorten consultations and enable physicians to focus the limited available time on elucidating finer details. Second, screening of OSA in relevant groups may be optimized. For example, a two-stage screening protocol has been proposed, with the STOP-BANG questionnaire followed by level III polygraphy demonstrating promising results, particularly for moderate/severe cases (Pangerc et al., 2024). Third, the very slow process of establishing an OSA diagnosis could be streamlined with such technologies. Home-PSG has been shown to have a similar failure rates as in-lab PSG (Bruyneel et al., 2015) (real-time TM monitoring could potentially improve this number further) (Bruyneel et al., 2013), relatively high accuracy (Garg et al., 2014; Banhiran et al., 2014), and possible cost-reduction (Pelletier-Fleury et al., 2001). Similarly, novel monitors, e.g., devices assessing mandibular movements using ML, have shown results comparable to those of manually scored in-home PSG (Kelly et al., 2022). However, for some HSAT devices, correlation with PSG is high (Yalamanchali et al., 2013), but weighted accuracy substantially lower, limiting the utility for both diagnosis and screening, other than for possibly moderate/severe cases (Massie et al., 2022).

## Treatment initiation, adherence, and success

The current first-line gold-standard treatment for OSA is PAP. PAP effectively maintains airway patency by generating sufficiently high air pressure and has various positive downstream effects, such as improvement in daily functioning, sleepiness, and blood pressure, but its effect is limited by its usage (Gottlieb and Punjabi, 2020). It has been estimated that suboptimal PAP adherence (if defined as <4 h per night) is seen in 29%−83% of patients (Weaver and Grunstein, 2008). Three years after initiation of PAP treatment, only around 50% continue using it (Pepin et al., 2021a). Further complicating the issue, long-term protection of PAP against all-cause and cardiovascular mortality is not entirely homogeneous (Benjafield et al., 2025), possibly in part due to the various phenotypes and endotypes that constitute OSA (Zinchuk et al., 2017). Comorbidities of high clinical relevance (e.g., systemic hypertension, atrial fibrillation, coronary artery disease, stroke) are also common in OSA, constitute to different phenotypes, and are associated with varying mortality risks (Bonsignore et al., 2019; Chiang et al., 2017). Symptoms such as excessive daytime sleepiness have been associated with a higher risk of long-term cardiovascular outcomes in OSA (Mazzotti et al., 2019). Different OSA subtypes are also linked to varying patterns of PAP adherence. For instance, patients with diabetes mellitus or chronic obstructive pulmonary disease (COPD) tend to exhibit lower adherence, whereas those with concomitant hypertension show the opposite trend (Pepin et al., 2021a). In short, PAP alone, particularly without monitoring adherence/efficacy, is inadequate for a substantial proportion of patients (Pépin et al., 2022), and for many patients, alternatives and/or additions to PAP are needed.

Starting from consultation about diagnostic tests and recommend treatment, TM has been reported to be a cost-effective and largely comparable alternative to corresponding in-clinic routines (Lugo et al., 2019; Coma-Del-Corral et al., 2013; Fauroux et al., 2023). As at least half of important pieces of information, on average, provided by a healthcare practitioner are forgotten by the patient (Kessels, 2003; McCarthy et al., 2012), a TM-based solution may provide better and longer-lasting understanding of what is expected in the treatment and follow-up. It would be particularly useful for select subgroups and if instructions and explanations can be saved and reviewed later when needed, e.g., through dedicated apps. Similarly, studies suggest that TM-based PAP initiation is not inferior to in-lab PAP initiation (Fietze et al., 2022a; Contal et al., 2021). Cost-effectiveness may be also achieved, at least indirectly through reduced loss of productivity and transportation savings, in TM-based PAP follow-up (Isetta et al., 2015; Turino et al., 2017) and adherence assessment (found to be efficient while reducing labor costs) (Munafo et al., 2016). Based on extant studies, PAP adherence itself is at least comparable or even surpasses that attained with standard care (Coma-Del-Corral et al., 2013; Turino et al., 2017; Hoet et al., 2017; Fridriksson et al., 2023; Woehrle et al., 2018), although the type of TM appears to matter important, with interactive models likely being more effective than standardized information (Hwang et al., 2018; Pengo et al., 2018). Speaking of apps, a smartphone-based solution for self-monitoring of PAP treatment has been reported to have relatively high feasibility and usage rates, particularly so in PAP-naïve participants. Interestingly, those who used the app regularly were substantially older than those who did not, highlighting the difficulty in assessing who will benefit the most from specific treatment modalities (Isetta et al., 2017). It is also important to note, that it appears feasible to accommodate TM-based management for patients with limited technical know-how (Garmendia et al., 2018). Further, TM monitoring could aid in streamlining the identification and management of those that struggle with treatment, redirecting resources more efficiently between adherent patients and non-adherent patients, the latter necessitating more intensive interventions to reduce the risk of treatment termination (Texereau et al., 2024). As a more specific example, PAP non-adherent patients with residual excessive daytime sleepiness identified through TM may be offered non-PAP alternatives and potentially wake-promoting drugs (Steier et al., 2023; Randerath et al., 2022; Verbraecken et al., 2022; Randerath et al., 2021; Dauvilliers et al., 2020; Pepin et al., 2021b). TM for non-PAP devices are also being actively developed, e.g., sensors embedded within mandibular advancement devices (MADs), accurately measuring compliance and respiratory events (Dieltjens et al., 2013; Mohammadieh et al., 2025).

Beyond managing the OSA itself, TM may enable physicians to keep track of relevant comorbidities. For example, TM can provide early alert of serious cardiac events, e.g., through rapid increase in residual AHI (identifying Cheyne-Stokes respiration, associated with heart failure, atrial fibrillation, etc.) (Prigent et al., 2022). Extending further, multimodal TM monitoring may provide benefit in relevant comorbid conditions, e.g., increasing the number of daily steps (Murase et al., 2022), and possibly a more health-conscious lifestyle overall, indicated by improved sleep quality and quality of life (Pépin et al., 2019), although more substantial changes, such as lower body mass index or blood pressure have not been (consistently) reported (Murase et al., 2022; Pépin et al., 2019). All in all, TM allows for collection of rich daily information of various kinds, including residual respiratory events and treatment device malfunction, with potential to build longitudinal time series for sophisticated AI analyses and anomalies (Bottaz-Bosson et al., 2023).

Finally, in terms of perceived success and satisfaction with treatment, TM-based solutions appear at least non-inferior to traditional care (Fridriksson et al., 2023; Parikh et al., 2011; Berry et al., 2020). Importantly however, this aspect is complex to evaluate, and is heavily influenced by the quality of usual care, the length of follow-up, and other factors. Nevertheless, the positive findings are important corroborations of the validity of TM in OSA, e.g., because capturing and properly managing PROMs may improve PAP adherence (Cistulli et al., 2023).

## TM in multidimensional treatable traits-based and AI-driven management

Management of OSA is complex and multifactorial, as the patients are of higher age and with comorbidities (Bonsignore et al., 2019). In recent years, management based on TTs (quantifiable and treatable disease characteristics) (McDonald and Holland, 2024) has been proposed in various areas, including asthma (Gibson and McDonald, 2024), COPD (Cardoso et al., 2021), and lately also for OSA (Pardo-Manrique et al., 2024; Lisik and Zou, 2025a). The TTs paradigm in OSA emphasizes individualized management by identifying and targeting clinically relevant patient-specific characteristics. These include endotypic and phenotypic traits, behavioral factors, comorbidities, health literacy, patient engagement, and subjective needs or preferences. This precision-based approach thereby reduces therapeutic bias and improves clinical outcomes (Lisik and Zou, 2025a; Hrubos-Strøm et al., 2023). For instance, glucagon-dependent insulinotropic polypeptide (GIP)/glucagon-like peptide-1 (GLP-1) receptor agonists may represent a rational therapeutic option for patients with obesity and moderate-to-severe OSA (Lisik and Zou, 2025b). However, healthcare systems are economically stressed and the demand is ever-increasing (Goryakin et al., 2020), which renders a comprehensive focus (which in traditional settings requires substantial labor-costs with cross-disciplinary/specialist involvement etc.) even less feasible. We argue that TM, particularly coupled to AI, is a sensible approach to enable data-driven, and simultaneously patient-centered, TT-based OSA management.

**Figure 2:**
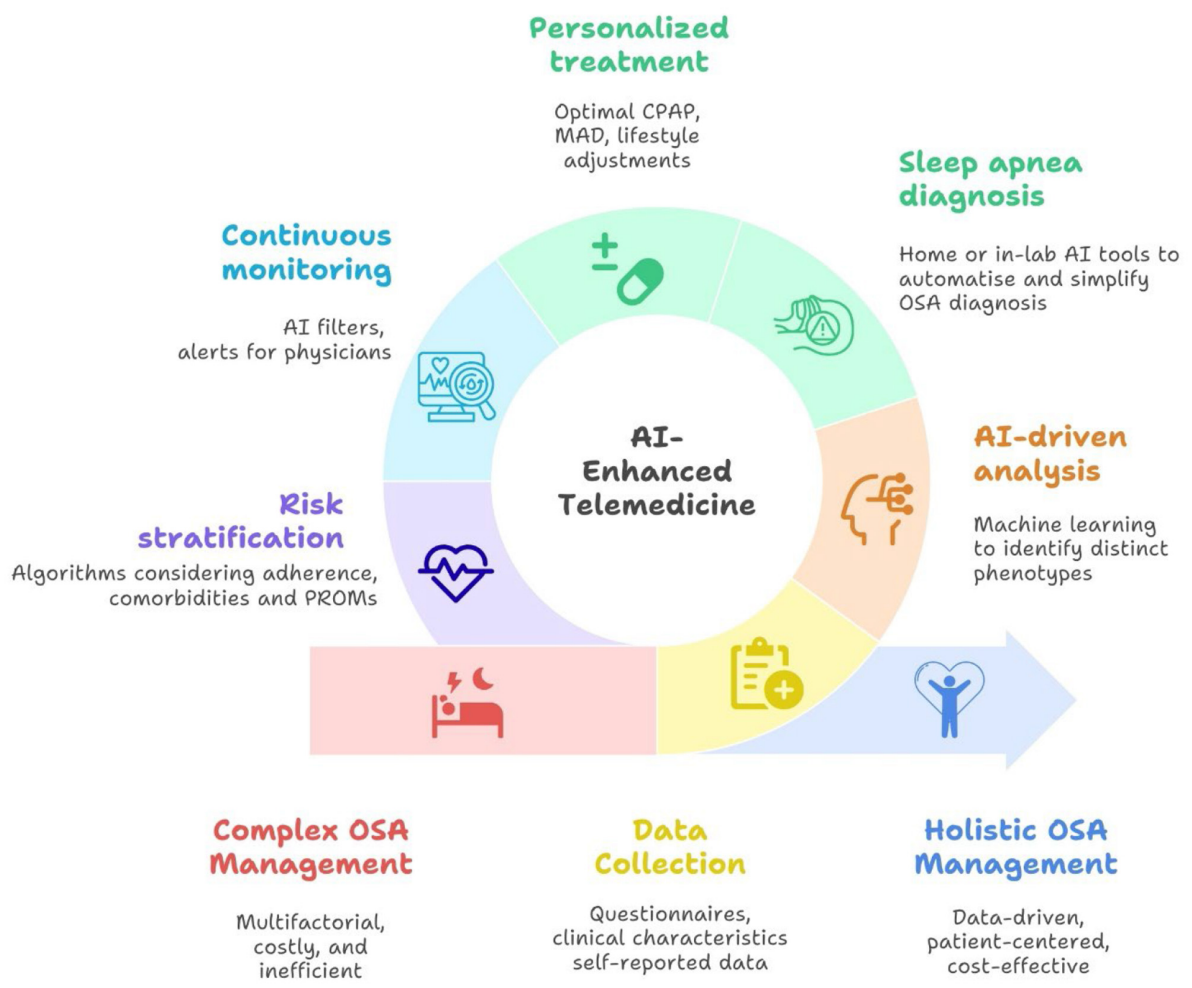
Conceptual framework for artificial intelligence (AI)-powered telemedicine-based management of obstructive sleep apnea (OSA). CPAP, continuous positive airway pressure; MAD, mandibular advancement device; PROM, patient-reported outcome.

In an ideal scenario, a seamless personalized protocol can be applied, with multimodal data, incorporating relevant comorbidities, lifestyle/anthropometric/risk factor information, as well as personal preferences and health goals—from screening/diagnosis to treatment initiation, optimization, and follow-up (Figure 2). In short, following the completion of questionnaires, HSAT, and in relevant cases in-lab PSG, a thorough phenotyping is conducted. This involves an explainable, deep machine learning–based cluster analysis that provides a clear rationale for patient segmentation (Belle and Papantonis, 2021). The analysis considers multidimensional patient features to identify the specific OSA subtypes and determine the most appropriate treatment approach such as PAP, MAD, or other alternatives. It also supports the adjustment of therapy and the monitoring of disease progression and risk factors. In parallel, risk stratification is performed with an explainable prediction model, to further weigh the risk of adverse outcomes and potential treatment modifications, based on the specific individual's data and not solely on the associations seen in a larger subgroup of patients. The prediction model can then be run iteratively based on the continuously collected patient data to assure that the optimal pathway is kept throughout the management journey. AI solutions, not least LLMs, may aid in synthesizing continuously collected patient data (both pertaining to the treatment, e.g., PAP usage, and relevant physiological metrics/comorbidities and subjective experience of disease) and efficiently filter out important deviations, reporting to treating physicians only when needed and thereby greatly reducing administrative burden. For example, a smartphone chat-based app may “keep up” with patients in an encouraging and natural manner about their treatment goals, adherence, and overall health, and alert the treating physician when needed.

Although various successful integrations of TM in OSA management are already used in practice, there are multiple “loose threads” and a more comprehensive focus is yet lacking. For example, it is known that co-morbid insomnia and sleep apnea (COMISA) necessitate a more multidimensional management strategy (including treatment of insomnia) (Sweetman et al., 2022). Cognitive behavioral therapy is the first-line treatment for insomnia (Riemann et al., 2023), but it can be financially and practically inaccessible for many patients (Feuerstein et al., 2017). Internet-based interventions are promising (Brooker et al., 2025), but arguably even more so AI-enhanced and app/phone-based alternatives (Gkintoni et al., 2025). With the already relatively common LLMs available today, it is very likely that such solutions will provide immense benefit at substantially reduced cost. Nevertheless, it is crucial to remain cautious of the fact that the optimal extent and form of TM most definitely differs from patient to patient. PAP therapy is a lifelong treatment that significantly improves daytime symptoms in patients with OSA. However, asymptomatic patients may be less motivated to adhere to therapy, as they perceive minimal changes in PROMs in their daily lives, which can contribute to declining compliance over time. The integration of TM with AI has the potential to enhance and sustain adherence by enabling personalized goal setting and delivering tailored supportive interventions (Verbraecken, 2021; Kaye et al., 2025). From a TT perspective, incorporating TM in those with comorbidities is arguably of extra potential, and with the wide availability of consumer technologies and capable smartphones, collecting rich longitudinal data is not a practical issue in many contexts. The benefits of synchronous TM are likely modest at best (Verbraecken, 2021; Kuziemsky et al., 2019). However, “meeting” and interacting with patients in the context of their daily lives, particularly through AI interfaces that can triage and integrate patient input, may represent one of the most promising avenues for these technologies in OSA management. Evidence for this is seen in the effectiveness of motivational feedback, which, albeit automated, provides direct and continuous feedback and ultimately direct patient engagement and understanding of treatment success.

## Limitations and future perspectives

TM in OSA holds promise of practical validity and utility, not least seen through the recent rapid implementation (Verbraecken et al., 2025). However, the field remains relatively new, and long-term studies are few and many details are yet to be elucidated. From a large perspective, there is not even consensus on the number of follow-up meetings (virtual *or* face-to-face) needed for optimal treatment outcomes (Pépin et al., 2025). Similarly, although many AHI alternative metrics have been investigated and found to provide predictive power to adverse outcomes, more prospective and generalizable studies are needed, and most certainly this is the case for specific subgroups (Azarbarzin et al., 2025). In general, more studies are needed to elucidate the most appropriate implementation of TM in specific subtypes of OSA. AI, not least LLMs, while highly promising, carry the risk of alienating patients if trust is not instilled, through proper explainability/transparency and an optimal balance between physician and machine interaction. There are also legal and practical/financial challenges. For example, TM use was not boosted during the COVID-19 pandemic in Europe, possibly due to dominance of German centers with largely traditional practice, insufficient staffing, and data security/privacy concerns (Grote et al., 2020). Overnight PSG may include personal information such as video and audio recordings, therefore, analyzing this data using AI requires careful consideration due to privacy concerns. Thus, a shift is needed in attitude and routines, but also in juridical restrictions of data-sharing, not only between patients and their healthcare providers, but also between institutions performing research, as the vast data collected with TM is nothing short of a gold mine for AI-based analyses and important discoveries necessitating large-scale data. It has also been shown that TM of high quality necessitates efforts to set up proper infrastructure and workflows (sometimes including of time and practical labor, e.g., standardization in data handling/processing) (Bottaz-Bosson et al., 2023), and prospective well-designed studies are warranted to elucidate which forms of TM, and in which contexts, do the most good. Further, inequalities in access and competence in using technological devices (the “digital divide”) may hamper TM-based efforts in some of the most needed subgroups (Sanders and Scanlon, 2021). Finally, for the foreseeable future, the intuition and comprehensive assessment of a clinician will remain the centerpiece in all OSA management. Thus, while a useful tool for streamlining processes and collecting/sorting information, TM should remain a co-pilot to the physician.

## Concluding remarks

TM, particularly coupled with AI, holds great potential for reducing costs, shortening waiting time for patients, and ultimately (and most importantly) to provide optimal management of OSA for more patients, with consideration of individual variability in diagnosis, comorbidity patterns, and treatment response. In accommodating the commonly seen picture of complex comorbidity patterns and lifestyle/sociodemographic factors influencing disease, treatment response/adherence, and adverse outcomes, synergy may be achieved, and overall health may be improved of affected individuals. TM may also act as an important catalyst in research through the immense data collected. However, more long-term studies and studies focusing on specific subgroups of patients are warranted, and for the foreseeable future, clinicians need to remain in control of managing this multifactorial and complex disease (Pittman et al., 2024).

## Data Availability

The original contributions presented in the study are included in the article/supplementary material, further inquiries can be directed to the corresponding author.
